# Evaluation of zinc-oxide nanocoating on the characteristics and antibacterial behavior of nickel-titanium alloy

**DOI:** 10.1590/2177-6709.25.4.051-058.oar

**Published:** 2020

**Authors:** Shaza M. Hammad, Noha A. El-Wassefy, Marwa Sameh Shamaa, Ahmed Fathy

**Affiliations:** 1 Mansoura University,Faculty of Dentistry, Department of Orthodontics (Mansoura, Egypt).; 2 Mansoura University, Faculty of Dentistry, Department of Dental Biomaterials (Mansoura, Egypt).

**Keywords:** Friction resistance, Antibacterial agents, NiTi orthodontic wires, ZnO nanoparticles

## Abstract

**Objective:**

To investigate the effect of ZnO nanocoating on mechanical properties of NiTi orthodontic wires and antibacterial activity.

**Methods:**

0.016 x 0.022-in NiTi orthodontic wires were coated with ZnO nanoparticles using an electrochemical deposition method with three electrodes system in 0.1M Zn(NO_3_)_2_. Mechanical properties and frictional resistance of the coated wires were investigated using an universal testing machine. Antibacterial effect of ZnO coating was also investigated.

**Results:**

A stable adhered ZnO nanocoating on NiTi wires was obtained. The coated wires have a significant antibacterial activity against *S. aureus*, *S. pyogens* and *E. coli*, and a reduction of frictional forces by 34%.

**Conclusion:**

ZnO nanocoating may improve the antibacterial effects of NiTi wires and reduce the frictional resistance. Coating may be implanted in orthodontic practice for faster and safer treatment.

## RESUMO

**Objetivos:**

Avaliar o efeito do nanorrevestimento de óxido de zinco (ZnO) sobre as propriedades mecânicas e propriedades antibacterianas de fios ortodônticos de NiTi.

**Métodos:**

Fios 0,016” x 0,022” de NiTi foram revestidos com nanopartículas de ZnO por meio de um método de deposição eletroquímica com um sistema de três eletrodos a 0,1M Zn(NO_3_)_2_. Uma máquina universal de testes foi utilizada para avaliar as propriedades mecânicas e a resistência friccional dos fios revestidos. Além disso, também foram analisadas as propriedades antibacterianas do revestimento de ZnO.

**Resultados:**

Obteve-se uma aderência estável das nanopartículas de ZnO sobre os fios NiTi. Os fios revestidos apresentaram atividade antibacteriana significativa contra *S. aureus*, *S. pyogens* e *E. coli*, e apresentaram uma redução de 34% na força de atrito.

**Conclusão:**

O revestimento com nanopartículas de óxido de zinco pode melhorar as propriedades antibacterianas e reduzir a resistência friccional dos fios de NiTi. Assim, o revestimento dos fios pode ser utilizado na Ortodontia visando tratamentos mais rápidos e seguros.

## INTRODUCTION

Nickel-titanium (NiTi) wires have unique properties compared with other types of wires. NiTi wires can generate light forces in a large range of action, so they are considered to be the ideal orthodontic archwires for the initial stage of comprehensive orthodontic treatment. The ability of the wire to slide along the bracket is essential for proper alignment and leveling in this stage.^1^ However, the main disadvantage of NiTi wires are surface roughness and high friction coefficient, which result in high frictional resistance.^1^ ^,^ ^2^ Consequently, higher orthodontic forces would be needed to overcome resistance to sliding and to achieve the desired tooth movement.^2,3^ Such excessive forces can increase the treatment duration, raise the risk of anchorage loss, undesirable tooth movement and root resorption.^4^ ^,^ ^5^

 The overall resistance to sliding is the sum of frictional resistance and binding. Binding occurs when contact points are formed between the edges of the bracket and wire, and when the angle between them exceeds the critical amount.^6^ Resistance to sliding is affected by some factors including wire and bracket material and surface characteristics. Various techniques are proposed to overcome resistance to sliding, including the use of different alloys, surface treatment, altering size and shape of the wire and bracket, and coating with different materials such as Teflon, inorganic fullerene-like nanoparticles of tungsten disulfide and carbon nitride film.^6,7,8^ The friction present during sliding mechanics represents a clinical challenge to the orthodontists because high level of fiction may reduce the effectiveness of the mechanics, decrease tooth movement and further complicate anchorage control.^9^ Reduction of friction between bracket and archwire can improve the orthodontic forces up to 50% and significantly facilitate tooth movement. It is also assumed to decrease treatment duration and the risk of apical root resorption.^10^

On the other hand, contact between orthodontic wires and brackets provides additional sites for microorganism binding and colonization.^11^ Demineralization of enamel and formation of white spot lesions (WSLs) are one of the most common side effects in fixed orthodontic treatments, with an estimated prevalence of 38% in the first six months and 50% at the end of the fixed orthodontic therapy, and may persist 5 years after the appliance removal. The major responsible factor for the formation of WSLs and dental caries is *Streptococcus* species.^12^ ^,^ ^13^

Many previous studies have investigated the antibacterial characteristics of coated orthodontic wires with different agents, including a photocatalytic titanium oxide (TiO_2_) with silver and copper oxide nanoparticles.^13^ ^,^ ^14^

This study aimed at coating NiTi wires with ZnO nanoparticles by electrochemical deposition. In this process, a thin and tightly adherent coating of metal oxide was deposited onto the surface of a conductor substrate by simple electrolysis in a solution containing the desired metal ion or its chemical complex. Electrochemical deposition has the advantage of providing corrosion resistance to the coated metals, thereby protecting the original material. In addition, the low cost and the ability to improve mechanical characteristics of coated metals are appreciated. It was also claimed that nanoparticles (NPs) may provide a new strategy for treating and preventing dental infections.^15^ The large surface area and high charge density of NPs enable them to interact with the negatively-charged surface of bacterial cells, resulting in enhanced antimicrobial activity.^16^ Moreover, NPs combined with polymers or coated onto biomaterial surfaces was found to exhibit superior antimicrobial properties in the oral cavity.^17^

The goal of this study was to deposit ZnO nanocoating on NiTi wires and evaluate the antibacterial resistance and the effect of nanocoating on frictional resistance of NiTi wires.

## MATERIAL AND METHODS

### Preparation of NiTi wires

Rectangular 0.016 x 0.022-in orthodontic wires commercially available (Ortho-Organizer, FL, USA) were ultrasonically cleaned in an absolute ethanol solution for 10 minutes at 37^o^C, followed by immersion of the wires in a 4 M potassium hydroxide KOH at 100^o^C for 30 minutes using magneto-agitator device.

### Coating wires with ZnO nanoparticles

Aqueous electro-deposition was performed using 0.1 M zinc nitrate Zn (NO_3_)_2_ that was prepared by adding 2.97 g of zinc nitrate to 100 ml of distilled water, then adding aqueous ammonia to the solution to make it alkaline, under vigorous mixing.

**Figure 1:**
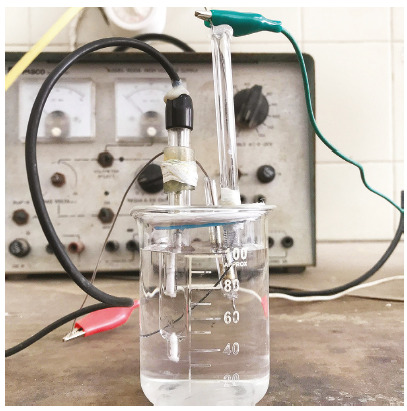
Three electrodes system in a single compartment cell.

Electro-deposition was performed in three electrodes system in a single compartment cell (Fig 1):

» Platinum disk (3.14 mm^2^) works as the counter electrode.
» NiTi wires act as the working electrode.
» Referencing electrode as SCC (saturated calomel electrode).

Potentiostat-galvanostat as power supply with applied potential ranging from - 0.91 to -1.1 volt for 2-3 minutes.

### Characterization of ZnO nanoparticles

The ZnO nanoparticles morphology and chemical composition of the particles were analyzed using scanning electron microscope and EDAX analysis.

### Antibacterial activity of the ZnO nanoparticles coated wires

Antibacterial activity of the coated wires was assessed against *Streptococcus pyogenes* (Gram-positive), *Staphylococcus aureus* (Gram-positive) and *E. coli* (Gram-negative). Twelve plates each containing 2 cc of nutrient agar were prepared under a septic condition then the plates were incubated for 24 hours at 37^o^C. 

Both coated and non-coated groups were tested for antibacterial activity. Twenty four wires were transferred to the plates, four plates for each type of bacteria. Bacterial growth inhibition was thus evaluated around the wires tested.

### Friction test

Friction measurements were developed to simulate sliding movements within a bracket system, and used for measuring frictional resistance using a Universal Testing Machine (UTM) (Lloyd LR 5K- England), composed by a frame for machine supporting, load cell for measurement of the forces, cross head, test fixtures and output devices. The machine was connected to a computer for force analysis and printing of the results. Twenty pieces of wire were prepared for friction test. To simulate the sliding of the tooth across the archwires, 0.022-in slot stainless steel brackets (Ortho-Organizer, FL, USA) were used. The wires were connected to brackets by elastomeric ligatures. Brackets were bonded to the metal bars using cyanoacrylate bonding agent, then the metal bars were attached to the base of the universal testing machine.

The wires were then pulled out from the brackets at a cross-head speed of 10 mm/minute with deflection limit of 3 mm and the load cell was calibrated between 0 and 10 N. After each test, the sample was replaced by another one, and finally all recorded data were collected and subsequently statistically analyzed.

### Statistical analysis

Data were analyzed with SPSS version 21. The normality of data was first tested with Shapiro-Wilk test. Continuous variables were presented as mean ± standard deviation (SD) for parametric data. The two groups were compared with Mann-Whitney test (non-parametric data), while ANOVA test was used to compare more than two groups (parametric data). Comparison between groups was performed by *post-hoc* LSD test.

## RESULTS

### Characteristics of the ZnO nanoparticles

**Figure 2:**
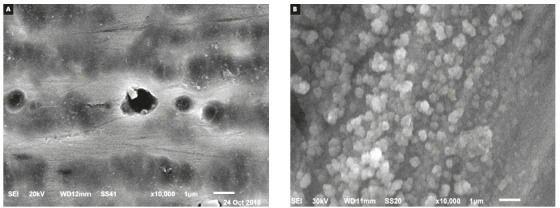
A) Photomicrograph of non-coated NiTi wires, B) Photomicrograph of ZnO nanocoating on NiTi wires.

**Figure 3:**
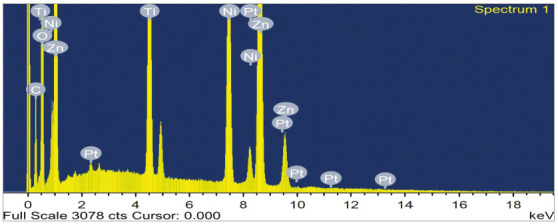
EDAX analysis of ZnO nanocoated NiTi wires.

**Figure 4:**
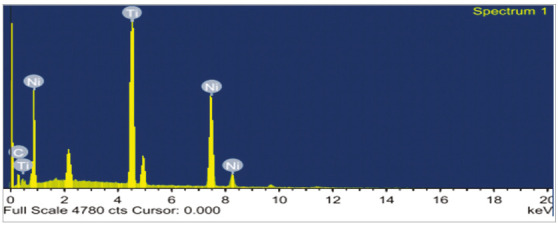
EDAX test for uncoated NiTi wires.

Scanning electron microscope of the ZnO nanoparticles coated wires demonstrated a homogenous layer of nanoparticles on the wire (Fig 2). EDAX analysis demonstrated the formation of ZnO nanoparticles on the wire surface (Zn = 20% by weight, O = 45% by weight, Ti = 7.15% by weight and Ni = 8.81% by weight), as shown in Figure 3 and Table 1. EDAX analysis of non-coated NiTi wires (Ni = 48.62% by weight, Ti = 40.91% by weight and C = 10.47% by weight) is show in Figure 4 and Table 2.

**Table 1:** Atomic% and weight% of the elements of ZnO coated wires.

Element	Weight%	Atomic%
C K	16.26	27.97
O K	45.92	59.29
Ti K	7.15	3.08
NI K	8.81	3.10
Zn K	20.14	6.36
P t L	1.72	0.18
Total	100.00	

**Table 2:** Atomic% and weight% of the elements of non-coated NiTi wires.

Element	Weight%	Atomic%
C K	10.47	34.13
Ni K	40.90	33.44
Ti K	48.62	32.43
Total	100	100

### Antibacterial activity test

While non-coated wires showed bacterial growth around the wires, coated wires showed no bacterial growth. None of ZnO-coated wires presented bacterial growth after incubation for 24 hours at 37^o^C regarding all types of bacteria used: *S. aureus* (Gram-positive), *S. pyogenes* (Gram-positive) and *E. coli* (Gram-negative). Inhibition zone was formed around all ZnO nanoparticles coated wires with all types of bacteria. Moreover, ZnO nanoparticles promoted more antibacterial effect on Gram-positive bacteria than Gram-negative bacteria (Table 3). 

**Table 3:** Mean diameter of inhibition zones around coated wires in different bacteria.

Type of bacteria	Mean	SD	Minimum	Maximum
*Staph. aureus*	4.25	0.49	3.70	4.80
*Strepto. pyogens*	6.25	0.64	5.50	7.00
*E. coli*	3.57	0.43	3.00	4.00
ANOVA Test	27.34			
P- value	<0.001*			

### Friction test

Table 4 shows that the presence of ZnO nanoparticles on the wires has decreased the mean frictional forces in the coated wires by 34%, compared with the non-coated wires (1.169 and 1.568 N, respectively). The coated wires showed a lower median frictional load (0.872 N) than uncoated wires (1.517 N), although no statistical significant difference could be found (*p*= 0.183).

**Table 4:** Comparison of Load at Limit (N) among coated and uncoated groups.

Load at Limit (N)	Coated group (n=15)	Uncoated group (n=10)	Mann-Whitney	P- value
Mean	1.169	1.568	1.33	0.183
SD	1.257	1.017		
Median	0.872	1.517		
Minimum	-0.78	0.26		
Maximum	3.98	3.73		

## DISCUSSION

NiTi archwire are the first choice for initial treatment as they provide light and constant forces for long periods without requiring several activations. However, they have great disadvantages due to the high friction coefficient. In addition, bacterial accumulation also occurs due to surface roughness. In this context, many attempts were done to overcome these problems and also to make orthodontics more esthetic. In this study, ZnO nanoparticles were used for coating of NiTi wires, characterized and investigated for their anti-bacterial properties, and friction resistance.

Chemical analysis of the coated wires demonstrated the formation of ZnO nanoparticles on the wire surface (O_2_= 59.29 atomic%, Zn = 6.36 atomic%, Ti = 3.08 atomic% and Ni = 3.10 atomic%). The present findings corroborate the results obtained by Kachoei et al.^14^ The EDS analysis of the coated wires confirmed that the wires consisted of nickel, titanium, zinc and oxygen. 

Surface topography of the ZnO nanocoating reveled homogenous layer of spherical shaped nanoparticles ranging from 40 to 60 nm in size on the wire surface. These results agree with Kachoei et al.^14^ Scanning electron microscope images showed spherical ZnO nanoparticles with particle size ranging from 25 to 30 nm. Behroozian et al.^19^ used SEM technique to evaluate surface pattern of ZnO nanoparticles deposition and showed the presence of spherically shaped ZnO nanoparticles on the wire. Kachoei et al.^20^ confirmed a uniform coating of spherical shaped ZnO nanoparticles on stainless steel wires with narrow size distribution ranging from 40 to 45 nm.

### Antibacterial characteristics

The incidence of WSLs and surface demineralization were noticed to happen in the first months of treatment and is initiated by *Staphylococcus* strains.^12^ ^,^ ^13^ Many attempts in previous studies were made to prevent WSLs, by brackets and wires coating with antibacterial agents. However, only few studies have explored the antibacterial effect of ZnO nanoparticles in orthodontic applications. The results of the present study revealed that ZnO nanoparticles had a significant antimicrobial activity against various bacterial strains: *S. pyogenes* (Gram-positive), *E. coli* (Gram-negative) and *S. aureus* (Gram-positive). ZnO nanoparticles have more bactericidal effect on Gram-positive bacteria than Gram-negative bacteria, according to the inhibition zones pattern noticed (Table 3). The antibacterial mechanism of NPs can be roughly divided into three types, although the specific mechanism of action is not yet clear. First, interacting with peptidoglycan cell wall and membrane, causing cell lysis; then, interacting with bacterial proteins and disrupting protein synthesis; and finally, interacting with bacterial (cytoplasmic) DNA and preventing DNA replication.^21^ ^,^ ^22^ ^,^ ^23^ The results of this study are in agreement with the results obtained by Ramazanzadeh et al,^18^ who studied the antibacterial effect of brackets coated with ZnO and CuO nanoparticles against *S. mutans,* and observed that the antibacterial effect of the coated brackets with ZnO-CuO and ZnO nanoparticles on *S. mutans* was excellent, since after two hours the bacterial count was reduced to zero. The coated brackets with ZnO nanoparticles ranked second, although in comparison with control group caused significant reduction of *S. mutans*, it could not reduce the population of *S. mutans* to zero even after 24 hours.^15^ Azam et al.^24^ compared the antibacterial activity of CuO, ZnO and Fe_2_O_3_ nanoparticles against Gram-positive (*S. aureus* and *P. aeruginosa*) and Gram-negative (*E. coli* and *Pseudomonas*) bacteria, and reported that ZnO nanoparticles have the best antibacterial effect and Fe_2_O_3_ nanoparticles exhibit the lowest activity. Although Cu nanoparticles have unique chemical, biological and physical properties and low cost of preparation, the rapid oxidation in air limits their application in orthodontics.^25^

It was suggested that the toxicity of antimicrobial nanoparticles is affected by many factors as dosage, type, particle size, distribution, duration of action, concentration and interaction with other compounds. Nanoparticles can enter the body and accumulate in the organs due to the small particle size. No study could be found; however, proving the cytotoxicity of nanoparticles on human beings. Although some few studies have been done to explain the cytotoxicity of antibacterial nanoparticles, there are no uniform indicators to standardize the toxicity of nanoparticles.^26^

### Mechanical properties

This study evaluated the effect of ZnO nanocoating of NiTi wires on frictional forces. The result showed a decreasing effect in friction resistance to sliding in the ZnO coated wires, compared to non-coated wires. The mean total frictional forces were estimated to be 1.169 N for coated wires and 1.568 N for uncoated wires, demonstrating a reduction of 34% after nanoparticles coating. These results coincide with the results obtained by Kachoei et al,^14^ who showed that the presence of ZnO nanoparticles coating on the wires has significantly decreased the frictional forces up to 21%. The frictional force was recorded as 1.227 N in the coated wires and 1.642 N for the non-coated wires. Also, Behroozian et al.^19^ studied the ZnO nanoparticles coating effect on the frictional resistance between ceramic brackets and orthodontic wires, and reported that the ZnO nanoparticles deposition had significantly decreased the frictional forces between brackets and stainless steel wires.

Samorodnitzky et al.^27^ found a significant decrease in kinetic and static frictional forces in NiTi and stainless steel orthodontic wires coated with inorganic fullerene of tungsten disulfide (IF-WS2) nanoparticles embedded in Co matrix up to 66%. They concluded that low friction nanocoatings could be applied for other biomedical purposes, as cardiovascular and orthopedic treatments. Wei et al.^28^ coated stainless steel orthodontic wires with CNx film and observed a significant reduction in the wire-bracket friction both in artificial saliva and in air.

Rapoport et al.^29^ and Cizaire et al.^30^ demonstrated the mechanism by which the frictional forces decrease between the wire and bracket after nanoparticles deposition. At first, nanoparticles act as a spacer, when the wire and bracket slots are parallel to each other, decreasing the surface sharpness and frictional forces. The frictional forces increased at slot edges, between the wire and the bracket slot angle. At that phase, some of the deposited particles flakes off the wires and their path of motion become more lubricious. The deposited nanoparticles are slowly flaked and washed out at interfacial areas under force application. It could also be stated that the deposition of ZnO nanoparticles on orthodontic wires can decrease frictional forces because nanoparticles protect the metallic wires against oxidation.^31^

Limitations of the study can be related to the fact that the exact coating thickness was not detected and also the variability of coating thickness with electrochemical deposition time, solution concentration and composition were not measured. 

## CONCLUSIONS

A unique coating on NiTi substrate was obtained using ZnO nanoparticles, which may have superior anti-bacterial effect against Gram-negative and Gram-positive bacteria and superior frictional performance. Nanoparticles coatings can be used in future orthodontic treatments.

## References

[1] Sivaraj A (2013). Comparison of superelasticity of nickel titaniumorthodontic arch
wires using mechanical tensile testing and correlating with electrical
resistivity. J. Int. Oral. Health.

[2] Kapila S, Angolkar PV, Duncanson MG, Nanda RS (1990). Evaluation of friction between edgewise stainless steel brackets
and orthodontic wires of four alloys. Am J Orthod Dentofac Orthop.

[3] Wichelhaus A, Geserick M, Hibst R, Sander FG (2005). The effect of surface treatment and clinical use on friction in
NiTi orthodontic wires. Dent. Mater.

[4] Redlich M, Katz A, Rapoport L, Wagner HD, Feldman Y, Tenne R (2008). Improved orthodontic stainless steel wires coated with inorganic
fullerene-like nanoparticles of WS (2) impregnated in electroless
nickel-phosphorous film. Dent Mater.

[5] Shirazi S, Kachoei M, Shahvaghar-Asl N, Sharghi R (2016). Arch width changes in patients with Class II division 1
malocclusion treated with maxillary first premolar extraction and
non-extraction method. J Clin Exp Dent.

[6] Burrow SJ (2009). Friction and resistance to sliding in orthodontics: a critical
review. Am J Orthod Dentofac Orthop.

[7] Drescher D, Bourauel C, Schumacher HA (1989). Frictional forces between bracket and arch wire. Am J Orthod Dentofac Orthop.

[8] Mirzakouchaki B, Shirazi S, Sharghi R, Moghimi M, Shahrbaf S (2016). Shear bond strength and debonding characteristics of metal and
ceramic brackets bonded with conventional acid-etch and self-etch primer
systems: an in-vivo study. J Clin Exp Dent.

[9] Rossouw EP (2003). Friction: an overview. Semin Orthod.

[10] Wei SB, Shao TM, Ding P (2011). Improvement of orthodontic friction by coating archwire with
carbon nitride film. Appl Surf Sci.

[11] Chun MJ, Shim E, Kho EH, Park KJ, Jung J, Kim JM (2007). Surface modification of orthodontic wires with photocatalytic
titanium oxide for its antiadherent and antibacterial
properties. Angle Orthod.

[12] Cheng L, Zhang K, Weir MD, Melo MA, Zhou X, Xu HH (2015). Nanotechnology strategies for antibacterial and remineralizing
composites and adhesives to tackle dental caries. Nanomedicine (Lond).

[13] Shah AG, Shetty PC, Ramachandra CS, Bhat NS, Laxmikanth SM (2011). In vitro assessment of photocatalytic titanium oxide surface
modified stainless steel orthodontic brackets for antiadherent and
antibacterial properties against Lactobacillus acidophilus. Angle Orthod.

[14] Kachoei M, Eskandarinejad F, Divband B, Khatamian M (2013). The effect of zinc oxide nanoparticles deposition for friction
reduction on orthodontic wires. Dent Res J.

[15] Magalhaes AP, Moreira FC, Alves DR, Estrela CR, Estrela C, Carrião MS (2016). Silver nanoparticles in resin luting cements: antibacterial and
physiochemical properties. J Clin Exp Dent.

[16] Cao W, Zhang Y, Wang X, Li Q, Xiao Y, Li P (2018). Novel resin-based dental material with anti-biofilm activity and
improved mechanical property by incorporating hydrophilic cationic copolymer
functionalized nanodiamond. J Mater Sci Mater Med.

[17] Saafan A, Zaazou MH, Sallam MK, Mosallam O, El Danaf HA (2018). Assessment of photodynamic therapy and nanoparticles effects on
caries models. Open Access Maced J Med Sci.

[18] Ramazanzadeh B Jahanbin A, Yaghoubi M Shahtahmassbi N, Ghazvini K Shakeri M (2015). Comparison of antibacterial effects of ZnO and CuO nanoparticles
coated brackets against Streptococcus mutans. J Dent Shiraz Univ Med Sci.

[19] Behroozian A, Kachoei M, Khatamian M, Divband B (2016). The effect of ZnO nanoparticle coating on the frictional
resistance between orthodontic wires and ceramic brackets. J Dent Res Dent Clin Dent Prospects.

[20] Kachoei M, Eskandarinejad F, Divband B, Khatamian M (2013). The effect of zinc oxide nanoparticles deposition for friction
reduction on orthodontic wires. Dent Res J.

[21] Cao W, Zhang Y (2017). Development of a novel resin-based dental material with dual
biocidal modes and sustained release of Ag+ions based on photocurable
core-shell AgBr/cationic polymer nanocomposites. J Mater Science Mater Med.

[22] Zhang N, Weir MD (2016). Orthodontic cement with protein-repellent and antibacterial
properties and the release of calcium and phosphate ions. J Den.

[23] Chen R, Han Z (2017). Antibacterial activity, cytotoxicity and mechanical behavior of
nano-enhanced denture base resin with different kinds of inorganic
antibacterial agents. Dent Mater J.

[24] Azam A, Ahmed AS, Oves M, Khan MS, Habib SS, Memic A (2012). Antimicrobial activity of metal oxide nanoparticles against
Gram-positive and Gram-negative bacteria: a comparative
study. Int Journal Nanomedicine.

[25] Dizaj SM, Lotfipour F, Barzegar-Jalali M, Zarrintan MH, Adibkia K (2014). Antimicrobial activity of the metals and metal oxide
nanoparticles. Mater Sci Eng C Mater Biol. Appl.

[26] Song W, Ge S (2019). Application of antimicrobial nanoparticles in
Dentistry. Molecules.

[27] Samorodnitzky-Naveh GR, Redlich M, Rapoport L, Feldman Y, Tenne R (2009). Inorganic fullerene-like tungsten disulfide nanocoating for
friction reduction of nickel-titanium alloys. Nanomedicine.

[28] Wei SB, Shao TM, Ding P (2011). Improvement of orthodontic friction by coating arch wire with
carbon nitride film. Appl Surf Sci.

[29] Rapoport L, Leshchinsky V, Lapsker I, Volovik Y, Nepomnyashchy O, Lvovsky M (2003). Tribological properties of WS2 nanoparticles under mixed
lubrication. Wear.

[30] Cizaire L, Vacher B, Le-Mogne T, Martin JM, Rapoport L, Margolin (2002). Mechanisms of ultra-low friction by hollow inorganic
fullerene-like MoS2 nanoparticles. Surf Coat Technol.

[31] Friedman H, Eidelman O, Feldman Y, Moshkovich A, Perfiliev V, Rapoport L (2007). Fabrication of self-lubricating cobalt coatings on metal
surfaces. Nanotech.

